# Undergraduate Public Health Education: Alternative Choices within the BSPH Degree

**DOI:** 10.3389/fpubh.2014.00127

**Published:** 2014-08-26

**Authors:** Karen Perrin, Laura Merrell

**Affiliations:** ^1^College of Public Health, University of South Florida, Tampa, FL, USA; ^2^Department of Community and Family Health, College of Public Health, University of South Florida, Tampa, FL, USA

**Keywords:** bachelors of public health, curriculum, blended degree, lessons learning, scheduling

## Abstract

The University of South Florida’s College of Public Health has been in existence since 1984. After many years of only offering a public health minor, a Bachelors of Science in Public Health was offered. This article explores the creation, development, scheduling, and lessons learned of this degree.

## Background

The University of South Florida (USF) is an urban, state, level one research institution located in Tampa, FL, USA with an enrollment of approximately 36,000 undergraduate and 11,000 graduate students. In 1984, the USF College of Public Health (COPH) was established with an initial enrollment of just 100 students. The College is fully accredited by the Council on Education for Public Health (CEPH), consists of five separate academic departments (Community and Family Health, Environmental and Occupational Health, Epidemiology and Biostatistics, Global Health, Health Policy and Management) and has graduated over 4,505 students in the past three decades. The COPH has close partnerships with other health-related colleges, including medicine, nursing, physical therapy, pharmacy, biomedical sciences, and social work. Current enrollment as of the summer of 2014 includes nearly 1000 Bachelor and 600 Master’s and Doctoral students. In 2011, the COPH became the first public university in Florida to house a Bachelor of Science in Public Health (BSPH) within an accredited school of public health. As of the spring of 2014, 714 students have graduated with the BSPH degree. In the academic year 2013–2014, 92% of students within the major graduated within 4 years. This degree attracts incoming freshmen, transfer students from community colleges, students wising to switch majors, and military veteran students making a career change. At any one time, the BSPH has on average 825 registered majors, far exceeding the 150 projected in at its creation.

Prior to the creation of the BSPH, the college only offered the General Public Health Minor (GPHM) in order to increase awareness about public health careers campus-wide and provide teaching opportunities and experience public health doctoral students. These courses served as general electives for the undergraduate students, and course management is handled within each COPH department. After offering the GPHM for several years, the Dean in conjunction with the faculty made the decision to initiate the BSPH. This decision was motivated by recent calls to increase public health workforce development. In particular, Healthy People 2020 calls an increase in the proportion of 4-year universities that offer general public health-related majors as well as those that are consistent with CEPH core competencies (1). By offering the BSPH, the COPH meets both of these objectives. In addition, considerable student interest for creating a major was expressed during an evaluation of the COPH minor.

The Office of Undergraduate Studies within the COPH is administered by the Assistant Dean and is housed in the Office of Academic and Student Affairs. This decision allowed the BSPH degree to remain a generalist degree with various elective courses offered by the five departments. The purpose of the BSPH is threefold: (1) to serve Florida by filling the critical need for public health workforce development; (2) to increase public health awareness among all majors by creating numerous electives and minors linked to departments; and (3) to serve as a conduit into careers in the health professions. Students who have graduated from this degree have found jobs in clinical health care facilities, government agencies, non-profit organizations, and health care corporations. The following discussion describes the curriculum, flexible scheduling, and accelerated degree options.

## BSPH Curriculum: Major and Minor

The BSPH requires 11 core courses (33 credit hours) and 4 elective courses (12 credit hours) in addition to the general University graduation requirements. The core courses were designed to cover foundational public health content and skills as well as integrate the CEPH core competencies into the curricula (a Healthy People 2020 objective) (1) (see Table 1). Although the BSPH has no formal concentrations, each COPH department offers elective courses specific to their discipline. Some departments have clustered the electives to establish a minor. The core courses introduce the students to the COPH departments.

**Table 1:** **Public Health Core Courses**.

Public Health Core Courses (33 required credit hours)	
PHC 3000 Introduction to Environmental and Occupational Health	HSC 4537 Medical Terminology
PHC 4000 Introduction to Epidemiology	HSC 4551 Survey of Human Disease
PHC 4050 Biostatistics in Society	HSC 4624 Foundations of Global Health
HSA 4101 Introduction to Public Health	HSC 4630 Understanding U.S. Health Care
HSC 4211 Health, Behavior, and Society	PHC 4810 Seminars (two three-credit courses = 6 credits)

There are seven minors, each consisting of five courses (15 credit hours) (see Table 2). These minors are promoted to all majors on campus. It is also possible for the BSPH students majoring to cluster their non-major electives to achieve a major and minor in public health. This option requires careful and accurate advising, since “double-dipping” (counting a course toward both the public health major and minor) is not allowed. Note that two minors offer certification opportunities.

**Table 2:** **Public Health Minors**.

Location	Minor
College	General Public Health
Department of Environmental and Occupational Health	Environmental and Occupational Health
Department of Global Health	Community Engaged Home Land Security and Emergency Management
	Infection Control (certification is available)
	Global Communicable Diseases
Department of Community and Family Health	Health Education (certification is available) Nutritional Science Maternal and Child Health (in development)

## Flexible Scheduling

By offering a curriculum with flexible scheduling and alternative calendar courses, the BSPH is designed to meet the needs of today’s students. Of 69 undergraduate courses offered by the COPH, 65% are housed at the department level and 35% at the college level. This serves a twofold objective: to allow students to become familiar with specific departments prior to selecting a concentration; and covers a portion of departmental funding of instructors while retaining oversight abilities at the college level. Currently, 52% courses are offered in the traditional classroom setting, 38% are offered online, and 10% courses are offered in both an online and classroom setting. In addition, there are three other types of flexible scheduling available for students. First, the alternative calendar allows students to take a three-credit course during spring break by attending class 9 h each day for five consecutive days for 45 contact hours. This course is called “Public Health Live On-Tour,” involves careful planning and coordination on behalf of both the instructors and College and University staff and administration. Each day begins with the two instructors and a maximum of 12 students meeting at the COPH, walking to the nearest bus stop, riding public transportation, and touring public health facilities across the county including an organic farm, solid waste facility, jail kitchen and tattoo salon inspection, biohazard disposal treatment site, and a tuberculosis treatment center. Second, Medical Terminology is a self-paced online course, so students may begin and complete the course at any time during the traditional 15-week format. Third, within the few weeks between fall, spring, and summer semesters, one online course is offered in a 3-week format called a “mini-mester.” This format allows students to focus their time on 12 course credits during the traditional semester without undue stress and then enroll in the mini-mester course, thus yielding 15 credits per semester for on-time graduation.

## Accelerated Degrees

In addition to flexible course scheduling, there are several accelerated degree options available for completion of the BSPH degree. First, local community college partnerships allow the opportunity to share courses between themselves and USF. In one example, the COPH shared the course materials for a personal wellness course with instructors at a rural community college. This arrangement saved their instructors time in creating a course from scratch and introduced their students to facets of public health, which they would not otherwise have had access to. In another example, an online lower level public health course allows community college students to make connections with COPH prior to completing their associate degree by becoming familiar with USF through summer semester and a personal connection with faculty members. This option creates a pipeline directly to the BSPH, so that community college transfer students do not lose their way on the large university campus.

In addition, the AS to BS degree allows students currently working in health care with a related associate degree (e.g., sonogram and radiology technician, dental hygiene technician, or surgical technician) to obtain a bachelor degree. A similar degree is the Bachelor of General Studies (BGS). This degree is attractive to working adults who may have obtained a collection of undergraduate credits but no associate or bachelor degree. These students have an accumulation of college transcripts and a desire to complete a bachelor degree. With careful review, advisors transfer as many credits as possible into required course categories and then students select a concentration, such as public health, hotel management, criminology, etc. Within a few semesters, these working adults may fulfill their dream of obtaining a bachelor degree.

Third, there are two BS to Master’s of Public Health degree options. The first BS to MPH is housed in the university’s Honors College and allows students of any major to apply to Master in Public Health after completing 90 undergraduate credit hours. Upon admission, they are able to complete four graduate level courses (12 credits) and thus fulfill their required upper-level electives for their bachelor’s degree as well as the core courses for their MPH degree. This overlap saves time as well as money since the 12 credits are billed at the undergraduate tuition rate. The second BS to MPH involves collaboration between the College of Arts and Science, Department of Geography, and the COPH Department of Environmental and Occupational Health. Like the first example, four courses (12 credits) overlap and count as undergraduate and graduate credits. The differences between the two accelerated degrees are related to the focus area. Both accelerated degrees required attentive and frequent advising with the student, both colleges and the registrar’s office.

## Lessons Learned

Since the BSPH was approved in February 2011, several valuable lessons have been learned related to the curriculum, flexible scheduling, and accelerated degree options. First, the curriculum needs to stay fresh and current. Generally, since most of the BSPH students are from the millennial generation, they enjoy a tech savvy learning environment. The flipped classroom model is proven successful when using teaching techniques in which the students study the didactic information prior to coming to class. This method allows the classroom time to focus on applying the information to practice through discussion, critical thinking, and case studies. This “read, reflect, and apply” method is appreciated by students as well as faculty. Second, flexible scheduling is appreciated and sought after by all types of students including the working students, athletes, active duty military, and students with families. By offering courses in the classroom and online allows students to learn in their preferred style. The alternative calendar courses and flexible schedule formats are appealing to students attempting to juggle complex work and family schedules. Third, although the accelerated degree options remain available for the BSPH students, these accelerated degrees are not as popular as once thought. The primary reason is because the students do not find out about the accelerated formats and thus complete their upper-level electives prior to reaching 90 credit hours. When this occurs, the accelerated format loses the time and cost-saving advantages. Also, some students do not wish to be locked into receiving their undergraduate and graduate degree from the same college at the same university. However, the students who discover and take advantage of the accelerate degree options report being pleased with the benefits.

In conclusion, it has been useful to try new teaching methods, scheduling, and new course topics. Even though not every new idea has been successful, it does show willingness toward creative and innovative education. However, even though repeating the same model for decades shows a consistent message to faculty and students, it does not keep pace with the ever-changing public health disciple.

## Conflict of Interest Statement

The authors declare that the research was conducted in the absence of any commercial or financial relationships that could be construed as a potential conflict of interest.

## References

[1] U.S. Department of Health and Human Services. Public Health Infrastructure [Internet]. Healthy People 2020 Objectives. Available from: http://healthypeople.gov/2020/topicsobjectives2020/objectiveslist.aspx?topicId=35

